# Microbial and Natural Metabolites That Inhibit Splicing: A Powerful Alternative for Cancer Treatment

**DOI:** 10.1155/2016/3681094

**Published:** 2016-08-16

**Authors:** Nancy Martínez-Montiel, Nora Hilda Rosas-Murrieta, Mónica Martínez-Montiel, Mayra Patricia Gaspariano-Cholula, Rebeca D. Martínez-Contreras

**Affiliations:** ^1^Laboratorio de Ecología Molecular Microbiana, Centro de Investigaciones en Ciencias Microbiológicas, Instituto de Ciencias, Benemérita Universidad Autónoma de Puebla, Edificio IC11, Ciudad Universitaria, 72570 Colonia San Manuel, PUE, Mexico; ^2^Laboratorio de Bioquímica y Biología Molecular, Instituto de Ciencias, Benemérita Universidad Autónoma de Puebla, Edificio 103H, Ciudad Universitaria, 72550 Colonia San Manuel, PUE, Mexico; ^3^Posgrado en Ciencias Químicas, Benemérita Universidad Autónoma de Puebla, Edificio 105 I, Ciudad Universitaria, 72570 Colonia San Manuel, PUE, Mexico

## Abstract

In eukaryotes, genes are frequently interrupted with noncoding sequences named introns. Alternative splicing is a nuclear mechanism by which these introns are removed and flanking coding regions named exons are joined together to generate a message that will be translated in the cytoplasm. This mechanism is catalyzed by a complex machinery known as the spliceosome, which is conformed by more than 300 proteins and ribonucleoproteins that activate and regulate the precision of gene expression when assembled. It has been proposed that several genetic diseases are related to defects in the splicing process, including cancer. For this reason, natural products that show the ability to regulate splicing have attracted enormous attention due to its potential use for cancer treatment. Some microbial metabolites have shown the ability to inhibit gene splicing and the molecular mechanism responsible for this inhibition is being studied for future applications. Here, we summarize the main types of natural products that have been characterized as splicing inhibitors, the recent advances regarding molecular and cellular effects related to these molecules, and the applications reported so far in cancer therapeutics.

## 1. Introduction

In eukaryotes, coding regions of the genome called exons are interrupted by noncoding sequences known as introns. During transcription, exons are identified while introns are removed from the immature mRNA (or pre-mRNA) to generate a mature and functional mRNA molecule. The mechanism responsible for this process corresponds to splicing and the machinery that performs this highly regulated event is the spliceosome, which is integrated by five small nuclear ribonucleoproteic particles (snRNPs) and more than 200 proteins that include auxiliary regulatory factors and components of other co- and posttranscriptional machineries [1]. During splicing, a series of RNA-RNA, RNA-protein, and protein-protein interactions are responsible for the decisions that determine which sequences will be included in the mature transcript [2]. Moreover, some sequences can be incorporated differentially into separated splicing events, leading to an increase in the coding potential of the genome by a process called alternative splicing.

## 2. Alternative Splicing and the Spliceosome

The general splicing mechanism involves the recognition of exon/intron boundaries in a sequence-dependent manner. In mammals, the 5′ end of the intron (5′ splice site or 5′ss) contains a characteristic TG, which recruits snRNP U1. On the opposite side, the 3′ end of the intron (3′ss) shows an invariant region called the branch point sequence (BPS), followed by a polypyrimidine-rich tract (pY-tract) and a conserved AG dinucleotide that indicates the end of the intron [3]. The recognition of the 3′ss involves the binding of SF1 to the BPS and the recruitment of the snRNP U2 auxiliary factor (U2AF) to the pY-tract and the AG dinucleotide. After the recognition of both exon/intron boundaries, an early complex is formed that commits pre-mRNA to undergoing splicing, where U2 snRNP is also recruited to the 3′ss. U2 snRNP recruitment to the pre-mRNA is one of the key steps that triggers additional interactions, leading to the formation of catalytic spliceosome complexes due to the incorporation of the tri-snRNP U4/U5/U6 within which numerous RNA rearrangements and modifications in protein composition contribute to complete a splicing cycle [2, 3].

Like most of the snRNPs, U2 is a ribonucleoproteic complex formed by 7 Sm proteins (which are common for spliceosomal snRNPs) and 17 specific proteins, being the largest snRNP [3]. Among the specific snRNP U2 components, two protein subcomplexes are found: SF3a and SF3b [3–5]. SF3a includes 3 subunits of 60, 66, and 120 kDa [6] while SF3b shows at least 8 specific subunits of 10, 14a, 14b, 49, 125, 130, 140, and 155 kDa [7]. Components of the SF3a and SF3b subcomplexes bind to sequences in the pre-mRNA tethering U2 snRNP to the BPS and the 3′ss. SF3b 155 is one of the most conserved subunits of U2 snRNP and it has shown the ability to bind splicing factors U2AF65 and p14 [3, 8]. Interestingly, this subunit has been related to the antiproliferative effect observed for some natural products that regulate the splicing mechanism and it results clear in the fact that targeting the spliceosome and modulating splice-site recognition could be relevant for the development of new therapeutic approaches, as will be further discussed.

## 3. The Role of Alternative Splicing in Human Disease

Over the past 10 years, the role of alternative splicing in human disease has been growing. When the human genome project was completed,* in silico* analysis predicted that 75% of the human genes underwent splicing [26] and that 15 to 50% of the genetic diseases were related to aberrant splicing events [27]. From this initial observation, several studies have linked splicing defects with specific genetic disorders. However, the full significance of the role in alternative splicing in human disease remains to be elucidated. Some diseases that have been linked to defects on splicing include dilated cardiomyopathy, autism spectrum disorder, spinal muscular atrophy, schizophrenia, cardiac hypertrophy, amyotrophic lateral sclerosis, and frontotemporal dementia [28]. In all these cases, the molecular insights related to the splicing defect that originates the disease have been dissected. The precise regulation of the splicing event varies for each pre-mRNA and for this reason it is time consuming to demonstrate the molecular mechanism that regulates the alternative splicing for each gene. Moreover, this regulation also depends on the cellular context, complicating the scene. In this regard, future efforts need to be developed in order to dissect the alternative splicing event that is related to each disease and the possible therapeutic tools that could be applied.

One specific group of diseases that have been related to splicing corresponds to different types of cancer and only recently the determinant role of splicing in cancer has been acknowledged [29, 30]. Several features of splicing events related to tumor progression have been reported and it is well documented that the alternative splicing of different pre-mRNAs is altered during oncogenic progression with the concomitant development of cancer features, like an increase in vascularization, cell proliferation, and invasion [31, 32]. The molecular hallmarks documented for several types of cancer have been recapitulated in an attempt to orientate future efforts towards cancer treatment through alternative splicing modulation [33–35]. Considering all this evidences, several studies have been oriented to modulate alternative splicing in order to treat cancer.

## 4. Microbial Metabolites That Regulate Splicing

Natural products have been traditionally sought from actinomycetes, filamentous fungi, and medicinal plants. In this regard, several derivatives of bacterial fermentation as well as their synthetic equivalents possess the ability to interact with components of the spliceosome. In some cases, the effect on splicing associated with these drugs is achieved through the direct regulation of the expression of genes that are relevant for cancer progression [36]. Dozens of small molecule effectors targeting the alternative splicing process have been identified and evaluated as drug candidates, including a natural product of* Pseudomonas* sp. number 2663 called FR901464 [9], natural products from* Streptomyces platensis* Mer-11107 that originated the group of Pladienolides [37], Herboxidiene [15], and Isoginkgetin [38]. These molecules and their derivatives have shown activity as splicing inhibitors and many of them demonstrated potent antiproliferative properties in human cancer cell lines, being in general less toxic to normal human cells [39].

### 4.1. FR901464 and Derivatives

**Figure 1:**
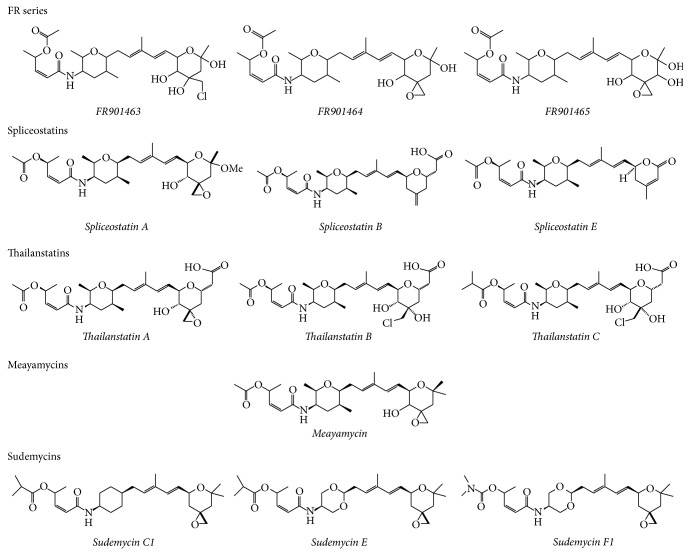
FR901464 and derivatives. The FR9014 series were isolated from* Pseudomonas* sp. number 2663 and constitute the first antiproliferative molecules associated with splicing inhibition. Spliceostatin A is a methylated derivative of FR901464. Spliceostatin B was also isolated from* Pseudomonas* sp. number 2663. Spliceostatin E was isolated from* Burkholderia* sp. FERM BP3421. Thailanstatins were recovered from* Burkholderia thailandensis* MSMB43. Meayamycin and Sudemycins are synthetic derivatives from the natural products depicted.

Spliceostatins are a group of compounds derived from the natural product FR901464, which was identified initially as an antitumor compound. In the original study, FR901463, FR901464, and FR901465 were isolated from the fermentation broth of* Pseudomonas* sp. number 2663 [9]. These 3 compounds are soluble in acetonitrile, chloroform, and ethyl acetate and poorly soluble in water and insoluble in hexane. They all show strong UV absorption at 235 nm distinctive of a conjugated diene, while the IR spectra indicated the presence of hydroxyl, ester, and a conjugated amide carbonyl (Figure 1). Initially, the three compounds from the FR9014 series mentioned before were tested for their biological activity. As a result, they all enhanced the transcriptional activity of SV40 in a CAT assay. Besides, they were all cytotoxic according to the MTT method in the following human adenocarcinomas: A549 lung cells, MCF-7 mammary cells, or HCT116 colon cells [9]. Moreover, the 3 compounds extended the life of mice bearing ascetic tumors, FR901464 being the one showing the most potent effect on the tumor systems assayed [9]. Furthermore, FR901464 induced characteristic G1 and G2/M phase arrest in the cell cycle and suppressed the transcription of some inducible endogenous but not housekeeping genes in M-8 cells. In this same cell line, internucleosomal degradation of genomic DNA showed the same kinetics corresponding to the activation of SV40 promoter-dependent cellular transcription, suggesting that a chromatin rearrangement occurs upon the treatment with the drug. Despite this effect in inducing viral gene promoters, it was observed that FR901464 reduces the mRNA levels of several endogenous genes, including c-Myc [9].

Spliceostatin A is a methylated and more stable derivative of FR901464 (Figure 1) and they both show similar activity [37, 40]; the synthesis and activity for both molecules have also been reported [41, 42]. Even when there are few studies on the molecular interactions that mediate the effect of Spliceostatin and related molecules, the antiproliferative effect of Spliceostatin has been associated with splicing and seems to be equivalent to the one registered after knocking down SF3b155 [10]. Using immunoprecipitation assays, further studies demonstrated that FR901464 and its methylated derivative Spliceostatin A inhibit pre-mRNA splicing both* in vivo* and* in vitro* by binding noncovalently to the SF3b subcomplex in the U2 snRNP. In the same study, the treatment with Spliceostatin A allowed the identification of immature forms of p27 by RT-PCR, suggesting that pre-mRNA molecules that have not been fully spliced are transported to the cytoplasm, inducing the translation of aberrant mRNAs [11].

Another analogous compound of FR901464 named Spliceostatin B was purified from the fermentation broth of* Pseudomonas* sp. number 2663 [43]. Spliceostatin B is soluble in DMSO, acetonitrile, acetone, water, chloroform, and dichloromethane. The structure of Spliceostatin B was determined using UV, IR, HR-MS, and NMR spectroscopic analyses, showing that it differs structurally from FR901464 at four points: the substitution of an epoxide group at C3 position with a terminal methylene moiety, the presence of a carboxyl moiety at C17 position, and the absence of two hydroxyl groups at C1 and C4 positions, respectively. These structural features are relevant for the biological function given the fact that it has been reported that loosing the C4 hydrogen bond donor decreases the cytotoxicity and that the C3 epoxide moiety is necessary for bioactivity [44]. The functional analog Spliceostatin B showed cytotoxic effect in three human cancer cell lines: HCT-116, MDA-MB-235, and H232A using the MTT method [45], but its activity was weaker than the one observed for FR901464 according to the IC values obtained [43] and in good correlation with the structural features just mentioned.

Other natural products considered Spliceostatin analogs were isolated from the fermentation broth of* Burkholderia* sp. strain FERM BP3421 [46]. Among these new molecules, Spliceostatin E exhibited good potency against multiple human cancer cell lines with IC_50_ values ranging from 1.5 to 4.1 nM. The structure of Spliceostatin E was elucidated by extensive spectroscopic studies and resulted structurally in less complex than Spliceostatins A and B [46]. Even when Spliceostatin E maintains the cytotoxic activity, the synthetic molecule showed no inhibition of splicing and it did not alter the structure of nuclear speckles [42].

It has been determined that the fr9 gene cluster is responsible for the biosynthesis of FR901464 in* Pseudomonas* sp. number 2663. The biosynthetic fr9 gene cluster spans a DNA region of approximately 81 kb and includes 20 genes (fr9A through fr9T). Using this information, a bioinformatic approach was conducted in order to identify other strains that could produce Spliceostatin-like metabolites. Using this comparative analysis while mining the genome of* Burkholderia thailandensis* MSMB43 elicited the identification of a biosynthetic gene cluster similar to fr9 that was named tst, referring to the Thailanstatin compounds it produces, which are functional analogs of Spliceostatins. The tst gene cluster spans a DNA region of 78 kb, which contains 15 ORFs designated tstA through tstR. The putative functions for the tst gene products were deduced by sequence comparisons with the FR9 proteins and with other bacterial homologs, where the most striking difference is the absence of the equivalent fr9S and fr9T genes from the tst gene cluster. A detailed analysis of this cluster suggested a possible biosynthetic route for Thailanstatins, which is similar to the one demonstrated for FR901464 and corresponds to a hybrid pathway involving a polyketide synthase and a nonribosomal peptide synthetase [47].

Consistent with the bioinformatic approach, Thailanstatins A, B, and C were isolated from the culture broth of* Burkholderia thailandensis* MSMB43 and they proved to be significantly more stable natural analogs of FR901464 [47]. These molecules are more stable because they lack a hydroxyl group found in FR901464 and they show an extra carboxyl moiety instead as revealed by the HR-MS, NMR, UV, and IR spectrometry. Thailanstatins possess the same linear polyketide-peptide framework observed in FR901464, but they lack a hydroxyl group at the C1 position while showing an extra carboxyl moiety at the C17 position. Thailanstatins B and C have a chloride substituent at the C3 position instead of the epoxide functionality observed for both Thailanstatin A and FR901464. Finally, Thailanstatin B possesses a dimethyl acetyl group at the distal end while Thailanstatin C shows an acetyl group instead [47].

During the biological tests performed, all Thailanstatins exhibited strong antiproliferative activities when tested in the following human cancer cell lines: DU-145 (prostate cancer), NCI-H232A (non-small-cell lung cancer), MDA-MB-231 (triple-negative breast cancer), and SKOV-3 (ovarian cancer), Thailanstatin A being the one showing the strongest effect. Moreover, the three compounds showed the ability to inhibit* in vitro* splicing and again Thailanstatin A showed the best result being as strong as FR941464 in inhibiting splicing [47]. A summary of the molecular effects depicted for FR9414 series and other splicing inhibitors is presented in Table 1.

Considering that the molecules just presented are structurally quite complex, this results in difficulty to accomplish their structural modification. However, synthetic derivatives have been generated (Figure 1), including Meayamycin and Sudemycins [48].

### 4.2. Pladienolides

**Figure 2:**
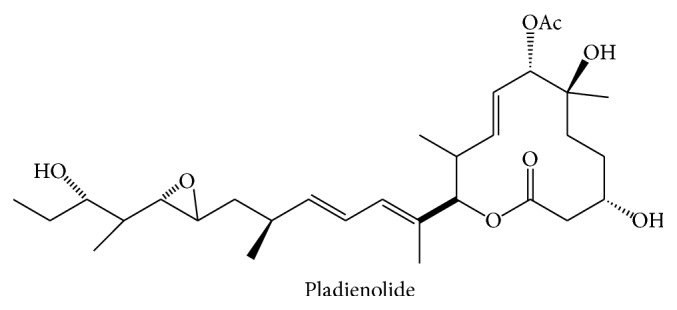
Pladienolide structure. Pladienolide is a 12-membered macrolide that possesses a long side chain at the carbon bearing lactone oxygen.

Pladienolide B is a macrocyclic lactone originally obtained from* Streptomyces platensis* Mer-11107, strain isolated from a soil sample collected in Kanagawa, Japan (accession number FERM P-18144, Bioconsortia Program Laboratory National Institute of Advanced Industrial Science and Technology, Japan). The compound deposited in the Open Chemistry Database (PubChem ID 52946850) has a molecular weight of 538.7132 g/mol (Figure 2).

Pladienolide B was initially identified in 2004, as part of a work that reported the isolation and structural and functional characterization of seven 12-membered macrocyclic compounds named Pladienolides A to G [37]. All these compounds displayed antiproliferative and tumor suppressive activities when assayed in cell culture and xenograft models, particularly Pladienolides B and D.

The initial extraction to isolate Pladienolides was performed with n-butanol from the fermentation broth of* Streptomyces platensis*. Further chromatography steps over Sephadex LH-_20_ and silica gel column were accomplished. The bioactive fractions recovered were subjected to preparative HPLC and each fraction containing pure Pladienolides was freeze-dried.

**Figure 3:**
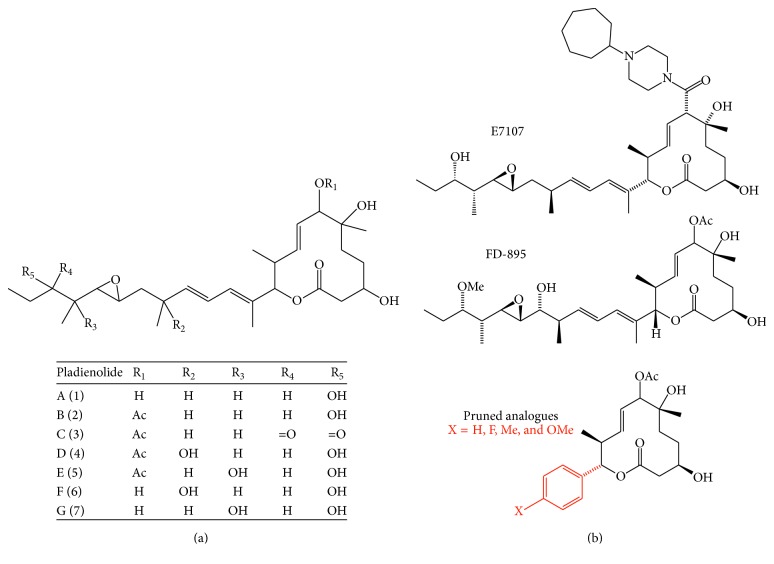
Pladienolide analogs. (a) General structure of Pladienolides A–G, which was determined by ^1^H, ^13^C NMR, MS, IR, and 2D NMR analyses. Radicals for each isoform are summarized in the table. (b) Different functional analogs have also been reported.

The chemical properties of Pladienolides were determined using spectroscopic methods. According to the physicochemical characterization of Pladienolides (A–G), they are soluble in methanol, acetone, n-butanol, ethyl acetate, and DMSO, but not in n-hexane, or poorly soluble in water. In all compounds there is a diene system evidenced by the UV absorption at 240 nm. The chemical structure of Pladienolides A (1), B (2), C (3), D (4), E (5), F (6), and G (7) (Figure 3) was determined by the analyses of NMR, MS, IR, and 2D NMR spectra. The carbonyl and hydroxyl groups were detected in the IR spectra. All Pladienolides are 12-membered macrolides possessing a diene unit and one epoxide moiety with a long side chain at the carbon that bears lactone oxygen [37]. Regarding their biological activity, Pladienolides have highly potent* in vitro* and* in vivo* antitumor activities with potential for use in anticancer therapy [20].

Pladienolide B has shown strong* in vitro* and* in vivo* antitumor activity and growth inhibitory effect against various cell lines, some of them being resistant to chemotherapeutic agents routinely used. Pladienolide B and some for their analogs induce cell cycle arrest at both G1 and G2/M [49].

A different study using Pladienolides was oriented to identify compounds that contribute of the adaptation of cancer cells to hypoxia using HIF-1, an HLH transcription factor involved in hypoxia adaptation in cancer cells. This approach consisted in searching inhibitors of hypoxia adaptation involved in the regulation of angiogenesis and anaerobic metabolism, considering that hypoxia-inducible genes are relevant for growth of cancer cells. The screening system consisted of the placental alkaline phosphatase (PLAP) gene reporter under the control of the human VEGF promoter containing the hypoxia-responsive element (HRE) that binds HIF-1. The reporter construction was transfected and the hypoxia-induced PLAP expression was analyzed in the U251 human glioma cells. Using a high throughput screening, Pladienolides were identified as inhibitors of hypoxia-induced PLAP expression when the cells were exposed to hypoxic conditions [50, 51]. The pure compounds were probed in their anti-VEGF-PLAP and antiproliferative activity, but only Pladienolides B and D showed a strong activity in both tests, with IC_50_ of 1.8 and 3.5 for Pladienolide B and of 5.1 and 6 nM for Pladienolide D. In other studies, Pladienolide B showed a potent tumor regression and inhibition of mouse xenograft acting at low-nanomolar concentrations (Table 2). The cell growth inhibition properties of Pladienolide B were identified in a study using a 39-cell line drug-screening panel and additional cell cycle analysis indicated that Pladienolide B blocks cell growth in both the G1 and the G2/M phase [13].

Pladienolide B is the most potent metabolite of* S. platensis* with antitumor activity, but the chemical synthesis of Pladienolide is complicated [52] and only three approaches have been reported for the synthesis of these unique macrolides [53–55]. On the other hand, the great amount of compound required for* in vivo* studies remains a significant challenge, due to the synthetic complexity inherent to this class of compounds. In an attempt to generate simple molecules that retain the biological activity of Pladienolides, several analogs have been developed. The more effective Pladienolide analogs for antitumor or anticancer application reported to date are Pladienolide D, E7107, and truncated-Pladienolide versions. Pladienolide D (16-hydroxylated pladienolide) is produced to a lesser extent than Pladienolide B on* S. platensis* Mer-11107. In order to facilitate the production of Pladienolide D, a biotransformation step of Pladienolide B into Pladienolide D was developed. In this alternative approach, the production of Pladienolide D was increased by 15-fold in the A-1544 strain of* S. bungoensis* by overexpressing the psmA gene, which encodes the Pladienolide B 16-hydroxylase (PsmA), responsible for the production of Pladienolide D [56]. Using a similar approach, the modified strain* S. platensis* Mer-11107 expressing the psmA gene from* S. bungoensis* A-1544 was obtained and in this case the production level of Pladienolide D was 10-fold higher [57]. Pladienolides B and D are promising candidates for further drug development because of their high efficacy and low toxicity; besides, their highly complex structure has been directed to the analog synthesis on a production scale [58].

E-7107 is a synthetic urethane derivative of Pladienolide D with activity against tumor cell lines and human xenografts [13]. E-7107 has a selective and potent antitumor activity in human tumor xenograft models such as human lung cancer LC-6-JCK, where E7107 caused complete tumor remission with poor toxicity. Moreover, E-7107 shows strong cell growth inhibitory activity against a large variety of human cancer cell lines (IC_50_ values range from 0.2 nM to 21.1 nM). Using an* in vivo* approach, E7107 produced significant tumor regression in a range of xenograft models. In this regard, animals with BSY-1 (breast), MDA-MB-468 (breast), LC-6-JCK (lung), NIH:OVCAR-3 (ovary) PC-3 (prostate), and WiDr (colon) xenografts were cured [25]. For this reason, E7107 rapidly advanced to Phase I clinical trials and the tests are currently in progress in Europe and the US (https://clinicaltrials.gov/ct2/show/NCT00459823?term=E-7107&rank=1). E7107 was tested in a Phase 1 clinical trial with patients with different types of solid tumors refractory to standard therapies, such as colorectal, esophageal, pancreatic, gastric, renal, and uterine, and was found to stabilize tumor growth [24, 59]. 40 patients received E7107 at doses from 0.6 to 4.5 mg/m^2^ as a 30-minute intravenous infusion on days 1 and 8 every 21 days. The MTD for E7107 using this schedule is 4.0 mg/m^2^ [24].

Finally, a different Pladienolide analog called FD-895 was isolated from* Streptomyces hygroscopicus* strain A-9561. FD-895 is a 12-membered macrolide antibiotic with a planar structure similar to Pladienolide D, but FD-895 has a hydroxyl group at the C-17 position and a methoxy group substituted for the hydroxy group at the C-21 position. FD-895 showed a cytotoxic activity against several types of cancer cells such as Adriamycin-resistant HL-60 [60]. In patients with chronic lymphocytic leukemia, FD-895 and Pladienolide B induced intron retention and spliceosome modulation. The cytotoxic effect of FD-895 involved the apoptosis induction in a caspase-dependent pathway [23].

### 4.3. Herboxidiene

Herboxidiene (GEXA1) is a polyketide [61] with the structure of a tetrahydrofuran with a residue of acetic acid and a conjugated diene [62]. These structural characteristics and its biological properties contributed to its name [63]. This compound was initially identified as a secondary metabolite from* Streptomyces chromofuscus* A7841. This compound was extracted with butanol and purified using HPLC. Further structural analysis was completed using HRFAB-MS and spectroscopic studies (RMN-^1^H y ^13^C).

**Figure 4:**
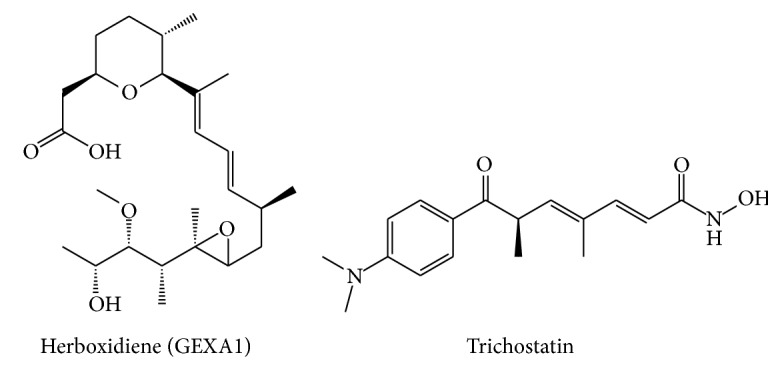
Herboxidiene structure. The characteristic structure of GEXA1 consisting of a tetrahydrofuran with a residue of acetic acid and a conjugated diene is shown in the left. This natural product was isolated from* Streptomyces* sp. The derivative Trichostatin is shown at right.

Initial applications for Herboxidiene included its herbicide activity [36]. In 2002, six molecules sharing similar structures were isolated from* Streptomyces* sp. and were called GEX1 (Figure 4). These compounds showed antibiotic and antitumor activities, GEX 1A being the molecule with the strongest antiproliferative effect, which was later identified as Herboxidiene [15, 64]. This cytotoxic activity seems to be related to cell cycle arrest in G1 and G2/M according to some* in vitro* experiments [65]. Besides the biological activities mentioned before, Herboxidiene has also shown activity as anticholesterol agent and as a potent splicing inhibitor.

Due to the multiple biological effects demonstrated for Herboxidiene, several groups have attempted the chemical synthesis of the compound. The first total synthesis was accomplished in 1999, where the relative and absolute configurations of Herboxidiene were confirmed [66]. Later attempts used several routes, including a stereochemical synthesis in 18 steps [67], an enantioselective synthesis in 16 steps [68], or an alternative synthesis in 16 steps with a global yield of 3.4% [69]. An additional chemical synthesis reported was performed starting from two chiral ketones derived from lactate in 14 steps with a global yield of 8% [70]. A total enantioselective synthesis of Herboxidiene was reported in 2014 and the obtained product showed a mild inhibitory activity on the spliceosome [71]. In that same year, the alternative synthesis of Herboxidiene and some other analogs like a Pladienolide-Herboxidiene hybrid was reported, where alternative splicing was efficiently modulated [72]. These observations supported the potential role of Herboxidiene analogs as drug candidates for cancer treatment.

Some molecules with pharmacological activities similar to those reported for Herboxidiene are Trichostatin and TMC-49A (Figure 4). These compounds have also shown anticholesterol activity. However, Herboxidiene has shown a stronger effect on lowering the amount of cholesterol in plasma by regulating the LDL (Low Density Lipoproteins) receptor [73].

It has been shown that Herboxidiene inhibits splicing due to its ability to bind SAP155, a component of the SF3b complex of the spliceosome, altering its functionality [65]. This was accomplished using tagged molecules of Herboxidiene [73].

The effect of Herboxidiene on the splicing of cancer-related genes has also been demonstrated for a couple of cases. For example, Herboxidiene inhibits the splicing of p27, generating an isoform that is unable to bind the E3 ligase, inducing the accumulation of p27, which is in turn free to recognize and block the E-Cdk2 complex responsible for the E3-mediated degradation of p27. Some of the evidence supporting the role of Herboxidiene in cancer regulation is summarized in Table 1.

### 4.4. Isoginkgetin

**Figure 5:**
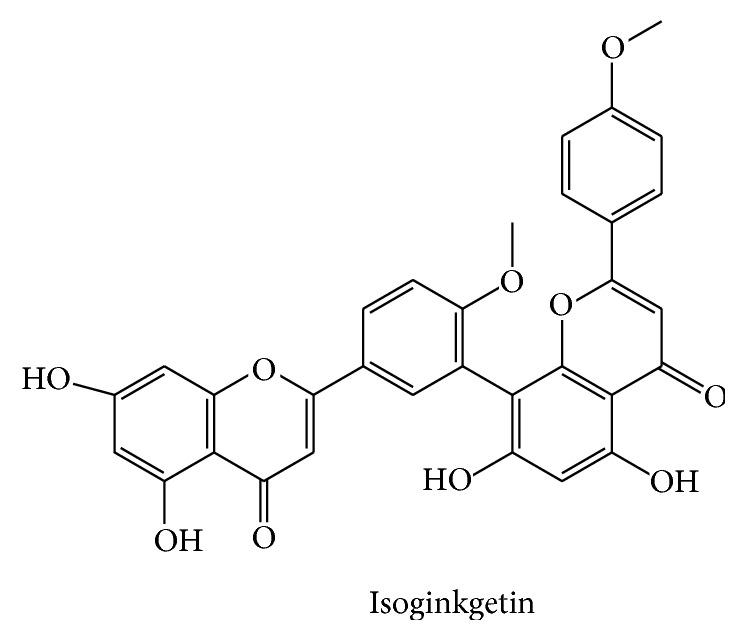
Structure of Isoginkgetin. The structure of the 7-*O-β-* d *-*glucopyranoside isolated from dried leaves of* Gingko biloba* is depicted.

Isogingketin (7-*O-β-* d *-*glucopyranoside) is a glycosylated biflavonoid (Figure 5) initially isolated from dried leaves of* Gingko biloba*, a medicinal plant long utilized in traditional eastern medicine. General extraction uses methanol and isolation was performed by column chromatography [38] while further characterization was completed using spectroscopic approaches including IR, UV, HR-FAB-MS, and NMR [74].

Baker and Ollis started to work on the isolation of a compound known as Ginkgetin in 1957; however, they were not able to succeed until later when the molecule was separated using a potassium salt using an approach developed in collaboration with Nakasawa [75]. When applying this technique, the recovered compound was an isomer of Ginkgetin and for this reason the molecule was called Isoginkgetin. From that moment, several studies have been performed in order to analyze the properties and applications of this natural compound.

After the initial isolation, Isoginkgetin has been obtained from other plants including* Dysoxylum lenticellare Gillespie* [76],* Chamaecyparis obtusa* [77],* Cephalotaxus koreana* [78], and* Cycas circinalis* [79] as well as from the fruits of* Capparis spinosa* [80],* Cyperus rotundus* [81],* Selaginella* [82], and* Podocarpus henkelii* [83].

The biological activity of several biflavonoid compounds has been analyzed in various studies due to its natural origin and its abundance in plants, especially in ferns. Demonstrated activities for the extract of* Ginkgo biloba* are miscellaneous, according to the following evidence. The anti-inflammatory activity has been related to the inhibition of arachidonic acid [84], the inhibition of COX-2 [85], SOD [86], cAMP phosphodiesterases [87], and the suppression of lymphocyte proliferation [88]. Other activities include the neuroprotector and cytoprotector effects when cells are exposed either to external or intrinsic factors like UV radiation [89] or the accumulation of *β*-amyloid in neurons [90, 91]. This extract has also been shown to increase adiponectin secretion [92] and the activity of AMP-kinases [93]. Considering these biological activities, Isoginkgetin has been a candidate compound to treat disorders like diabetes, Alzheimer's disease, and other neurodegenerative diseases [94].

In relation to the effect of Isoginkgetin in splicing, it has been shown that the molecule has the ability to inhibit splicing both* in vitro* and* in vivo* at similar concentrations (30–33 *μ*M). Isoginkgetin was identified as a splicing inhibitor using a cell-based reporter assay in HEK293 cells [95]. In the same study, it was suggested that the inhibitory effect possibly occurs due to the prevention of the stable recruitment of the U4/U5/U6 trismall nuclear ribonucleoprotein. Using HeLa cells, Isoginkgetin was applied to study* in vivo* mRNA dynamics, where an accumulation of intron-containing mRNAs was observed upon the treatment [96]. In this same cell line, Isoginkgetin mimics the effect of RNA exosome inhibition and causes accumulation of long human Telomerase RNA transcripts [97]. The effect of Isoginkgetin on splicing was also evaluated by studying the expression of interleukin 32 (IL-32) alternative isoforms, where IL-32*γ* isoform is overexpressed upon the treatment (Table 1), correlating with cell death in cell lines derived from thyroid cancer [19].

As observed for other splicing inhibitors, Isoginkgetin also shows antitumor activity, which has been related to the inhibition of metalloprotease MMP-9 production and to the increase of the inhibitors of metalloproteinase TIMP-1, resulting in a decrease of tumor invasion [18].

Further applications involving the use of splicing inhibitors can also expand our knowledge concerning the global regulation of gene expression. In a recent study, Isoginkgetin was coupled to a modified RNA-Seq method in order to provide a genome-wide insight into gene expression and to detect specific defects on splicing and transcription [96].

## 5. Mechanistic Insights

**Figure 6:**
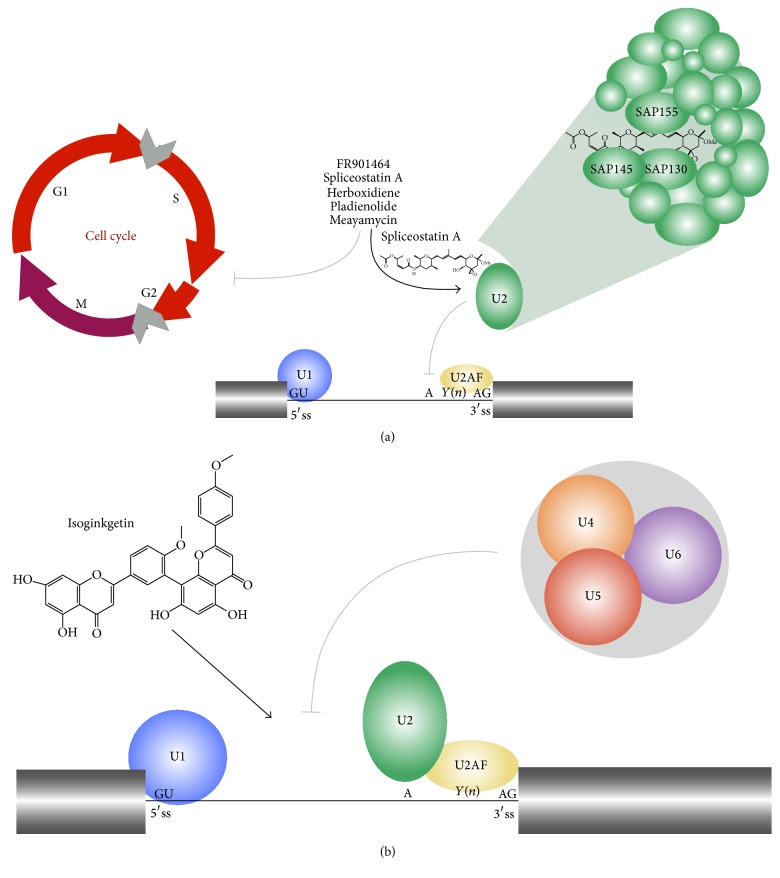
Molecular mechanism depicted for the natural products that inhibit splicing. (a) It has been demonstrated that FR901464, Spliceostatin A, Pladienolide B, Herboxidiene, and Meayamycin have the ability to block splicing by binding SAP130, SAP145, and SAP155 subunits of snRNP U2 (green). Besides, these natural products block cell cycle in G1 and G2/M transitions (gray arrows). (b) On the other hand, Isoginkgetin blocks splicing by inhibiting the incorporation of the tri-snRNP U4/U5/U6 complex to the spliceosome.

It has been demonstrated that Spliceostatin A, Pladienolide B, and Herboxidiene show the ability to interact directly with the spliceosome and that the molecular target for this molecule is the SF3b spliceosome subunit (Figure 6), a subcomplex of U2 snRNP [13]. The particular details observed for Pladienolide B are presented here.

Some studies have demonstrated that drug-treated human tumor xenografts can result in complete loss of the full-length mRNA for certain genes such as MDM2 in rhabdomyosarcoma cells. Pladienolide treatment causes an accumulation of unspliced or incompletely spliced pre-mRNAs and gives rise to fewer and larger nuclear speckles, the intranuclear sites where splice factors are stored [98]. Recently, it was demonstrated that splicing inhibition by Pladienolide B decreased phospho-Ser2 level [99] suggesting that the alteration of gene splicing may represent a suitable target for those drugs.

To identify the interaction between Pladienolides and a splicing protein, Pladienolide-tagged probes were used by modification of the acetoxy group at position 7 of Pladienolide B. Chemical tags included ^3^H-labeled, fluorescence-tagged, and photoaffinity/biotin- (PB-) tagged “chemical probes” (BODIPY-FL). The chemical probes were used in the VEGF-reporter gene expression and cell growth inhibition assays at low-nanomolar to submicromolar IC_50_. Pladienolide B blocks splicing and prompts nuclear export of intron-containing transcripts as observed by fluorescence microscopy, where the BODIPY-FL probe was concentrated in the nuclei of HeLa cells used as expression system overlapping with the signal obtained of splicing factor SC-35, a marker of nuclear speckles, where splicing factors are located [100]. Using immunoprecipitation experiments, the splicing proteins that interact with Pladienolide B were identified, such as 2,2,7-trimethylguanosine (TMG), Sm BB′&D1 protein, the U2 snRNP-specific protein U2B′′, spliceosome-associated protein 120 (SAP120, SF3a subunit 1), spliceosome-associated protein 155 (SAP155, SF3b subunit 1), and cyclin E. Therefore, U2 snRNP that functions at the 3′ splicing site (Figure 6) seemed to correspond to the target for Pladienolide B [21]. Further immunoprecipitation assays allowed the identification of SAP145 (SF3b subunit 2) and SAP130 (SF3b subunit 3) as Pladienolide B partners. In addition, it was reported that there is a direct interaction between the Pladienolide B probe and SAP130. The mechanism by which Pladienolide impairs* in vivo* splicing involves SF3b modulation by interacting with SAP130 in the SF3b complex, but it is also possible that Pladienolide B shows a partial interaction with SAP155 or SAP145. Besides, time- and dose-dependent disturbance of* in vivo* splicing with Pladienolide B results in the formation of enlarged “megaspeckles,” as observed when RNPS1 is overexpressed [101]. In a different approach using the CRISPR/Cas9 genome engineering system, it was demonstrated that the spliceosomal target of Pladienolide B is the SF3b1 subunit [102]. In the search of the mechanism by which Pladienolide B or E-7107 promote the formation of a defective spliceosome, it was found that E7 blocks ATP-dependent remodeling of U2 snRNP that exposes the branch point-binding region. Under this scenario, U2 snRNP fails to bind tightly to the pre-mRNA without disrupting the U2 particle or its association with SF3b [49, 103].

The use of the CRISPRCas9 system in HEK293T cells allowed the observation that some mutations in the subunits of the SF3b complex I promote higher levels of resistance to Pladienolide B. It was also found that R1074H mutation in SF3b1 could have a role in the resistance to Pladienolide [104]. In some reports, it has been proposed that mutations in SF3b1 are associated with numerous types of cancers such as acute myeloid leukemia, primary myelofibrosis, chronic myelomonocytic leukemia, breast cancer, chronic lymphocytic leukemia, and multiple myeloma [105, 106]. The sequence of the SF3b1 gene of 2087 patients with myelodysplastic syndromes (MDS) showed mutations in 20% of all of them, where the K700E mutation was the most frequently found [105]. Finally, the interaction of spliceosome modulators as Pladienolides with the SF3b complex leads to an imbalance in the splicing program in susceptible cells, which may induce apoptosis by changing the levels and/or ratios of essential (and aberrant) proteins in tumor cells [106].

## 6. Concluding Remarks

Alternative splicing is responsible for increasing the coding potential of the human genome and the implication of this mechanism in human health is starting to be elucidated. Future studies could analyze particular splicing events related to a specific genetic disease making the discovery of new drugs for the treatment of particular disorders possible. In the case of the treatment of cancer, available molecules that target the spliceosome have effectively reverted proliferation of different types of tumors with very low toxicity, suggesting that splicing modulation is an attractive target for cancer treatment. Moreover, considering that some of the splicing inhibitors recently discovered are natural microbial metabolites, it is possible to assume that there are several molecules with antitumor activity that remain to be discovered. Considering that the molecular mechanism related to particular types of cancer is being explored, it could be possible to develop new drugs that could be oriented to modulate a precise splicing event in order to treat a special genetic disease.

## Figures and Tables

**Table 1:** Molecular effects of different splicing inhibitors.

Splicing inhibitor	Cell line	Effect	Reference
FR9014 series	MCF-7	Induces G1 and G2/M arrest of the cell cycle	[9]
	HeLa	Inhibits the recognition of the branch point sequence	[10]
		Binding affinity to SAP145	[11]
		Arrest of SF3b	[12]
	MDA-MB-468	Interacts with SF3b subunit SAP145	[13]

Pladienolide	WiDr	Interacts with SF3b subunit SAP130	[13]
	HeLa	Interacts with SF3b. Remodeling of U2 snRNP to expose the branch point-binding region	[14]

Herboxidiene	Normal human fibroblast cell line WI-38.2	Induces G1 and G2/M arrest of the cell cycle	[15]
	HeLa	Causes arrest in G1 and G2/M phases and interacts with SF3b1 subunit SAP145	[16]

Trichostatin	WiDr	Interacts with SF3B subunit SAP130	[13]

Isoginkgetin	HT1080	Inhibition of Cathepsin K and MMP9	[17]
		Inhibits metalloproteinase MMP9 production and increases the synthesis of metalloproteinase inhibitor TIMP-1	[18]
	HEK293	Stimulates IL-8 expression	[19]
	Thyroid cancer	Increases expression of specific IL-32 isoforms and stimulates the expression of IL-8 and CXCR1	[19]

**Table 2:** Antitumor activity of pladienolides.

Molecule	Cancer type or cell line	Effect	Reference
Pladienolide B	Breast (BSY-1, MCF-7) Central nervous system (SF-539) Colon (HCT-116) Lung (NCI-H522, NCI-H460, A549, DMS273, and DMS114) Melanoma (OVCAR-3) Stomach (MKN74) Prostate (DU-145)	*Growth inhibition* Cell viability was evaluated with MTT and alamarBlue assay. The growth inhibitory activity corresponded to the concentration at which cell growth was inhibited to 50% of control growth (IC_50_). The strongest effect was observed for lung and breast cancer cell lines.	[20]

Pladienolide B	Anticancer drug-resistant cell lines: P388/CPT, P388/ETP, P388/CDDP, P388/VCR, HCT-116/5-FU, and MES-SA/Dx5	*Growth inhibition* Cell viability was evaluated with MTT and alamarBlue reagent and IC_50_ was determined. Pladienolide B showed differential strength depending on the cell line.	[20]

Pladienolide B	Human tumor xenografts: BSY-1, PC-3, OVCAR-3, DU-145, WiDr, and HCT116	*Antitumor* Cell suspensions of various human cancer cells were implanted subcutaneously into female or male BALB/c nu/nu mice. Tumor volume (TV) and relative body weight (RBW) were measured for 3 months after the treatment. Pladienolide B showed strong inhibitory or regressive activities against these xenografts.	[20]

Pladienolide B	WiDr and DLD1 human colorectal cancer cell lines	*Antiproliferative* Cells were incubated with 10 nM pladienolide B, 5-fluorouracil, taxol, or vincristine and then stained with propidium iodide. Pladienolide B caused a cell cycle arrest in both G1 and G2/M phases in a time-dependent manner according to FACS analysis.	[21]

Pladienolide B	Gastric cancer cell lines and primary cultured cancer cells from carcinomatous ascites of gastric cancer patients	*Antitumor* Using an MTT assay, the mean IC_50_ value was 1.2–1.1 nM for gastric, lung, and breast cancer cell lines. The mean IC_50_ value for primary cultured cells from the 12 cases studied was 4.9–4.7 nM. In xenograft models, the tumors completely disappeared within 2 weeks.	[22]

FD-895	Chronic lymphocytic leukemia	*Apoptosis* Peripheral blood mononuclear cells from CLL patients were exposed to 100 nM FD-895. Apoptosis was induced after 2 h exposure in an irreversible manner as measured by flow cytometry using a PI/DiOC_6_ assay.	[23]

E7107	Lung, breast, and colon tumors	*Antineoplastic* Tumor regression in different xenograft models: BSY-1 (breast), MDA-MB-468 (breast), LC-6-JCK (NSCLC), and NIH:OVCAR-3 (ovary). The efficacy was limited in 26 patients enrolled for a trial. Cell-cycle arrest at G1 and G2/M phases was observed by flow cytometry. Highest efficiency against tumors was accompanied by functional loss of Rb and an increase of the expression of p16 and cyclin E.	[24, 25]
